# S-Series Coelenterazine-Driven Combinatorial Bioluminescence Imaging Systems for Mammalian Cells

**DOI:** 10.3390/ijms24021420

**Published:** 2023-01-11

**Authors:** Genta Kamiya, Nobuo Kitada, Tadaomi Furuta, Takashi Hirano, Shojiro A. Maki, Sung-Bae Kim

**Affiliations:** 1 Department of Engineering Science, Graduate School of Informatics and Engineering, The University of Electro-Communications, Chofu, Tokyo 182-8585, Japan; 2 School of Life Science and Technology, Tokyo Institute of Technology, B-62 4259 Nagatsuta-cho, Midori-ku, Yokohama 226-8501, Japan; 3 Research Institute for Environmental Management Technology, National Institute of Advanced Industrial Science and Technology (AIST), 16-1 Onogawa, Tsukuba 305-8569, Japan

**Keywords:** bioluminescence, marine luciferase, coelenterazine, intensity pattern, color pattern, single-chain molecular strain probe

## Abstract

A unique combinatorial bioluminescence (BL) imaging system was developed for determining molecular events in mammalian cells with various colors and BL intensity patterns. This imaging system consists of one or multiple reporter luciferases and a series of novel coelenterazine (CTZ) analogues named “S-series”. For this study, ten kinds of novel S-series CTZ analogues were synthesized and characterized concerning the BL intensities, spectra, colors, and specificity of various marine luciferases. The characterization revealed that the S-series CTZ analogues luminesce with blue-to-orange-colored BL spectra with marine luciferases, where the most red-shifted BL spectrum peaked at 583 nm. The colors completed a visible light color palette with those of our precedent C-series CTZ analogues. The synthesized substrates **S1**, **S5**, **S6**, and **S7** were found to have a unique specificity with marine luciferases, such as R86SG, NanoLuc (shortly, NLuc), and ALuc16. They collectively showed unique BL intensity patterns to identify the marine luciferases together with colors. The marine luciferases, R86SG, NLuc, and ALuc16, were multiplexed into multi-reporter systems, the signals of which were quantitatively unmixed with the specific substrates. When the utility was applied to a single-chain molecular strain probe, the imaging system simultaneously reported three different optical indexes for a ligand, i.e., unique BL intensity and color patterns for identifying the reporters, together with the ligand-specific fold intensities in mammalian cells. This study directs a new combinatorial BL imaging system to specific image molecular events in mammalian cells with multiple optical indexes.

## 1. Introduction

Bioluminescence (BL) has been utilized in many bioassay systems as an efficient optical readout. Many BL systems are composed of the catalytic reaction of the substrate luciferin in the presence of luciferase and its cofactors [1]. For example, D-luciferin (LH_2_) is a common substrate of beetle luciferases and catalyzed by such as firefly luciferase (FLuc) and click beetle luciferase (CBLuc) in the presence of ATP, molecular oxygen (O_2_), and Mg^2+^ to generate green–red light (λ_max_ = 560~640 nm) [2,3]. On the other hand, native coelenterazine (nCTZ) is a common and natural luciferin of many marine luciferases. nCTZ is oxidized by the corresponding luciferase such as *Gaussia* luciferase (GLuc), *Renilla reniformis* luciferase (RLuc), *Oplophorus* luciferase (OLuc), and Metridia longa luciferases (MLuc) in the presence of O_2_. The natural luciferins for marine luciferases generally emit blue and green light (λ_max_ = 400~500 nm) [4,5]. Filling the deficient parts of the color palette in red should be a recent technological breakthrough in multiplex imaging systems [6].

To date, nCTZ has been targeted to improve the optical properties, such as intensity and color in BL imaging systems, through chemical modifications of the imidazopyrazinone backbone. For example, Furimazine is a successful substrate that has been used in various molecular imaging systems by pairing it with its specific luciferase, NanoLuc (shortly, NLuc), as a small subunit of the OLuc [7]. This Furimazine–NLuc combination has been adapted in many BL systems including protein-fragment complementation assay (PCA) [8], bioluminescence resonance energy transfer (BRET) [9,10], and various reporter-gene assays [11].

Many CTZ analogues have been synthesized to emit BL through its oxidative reactions with RLuc and its mutants [12,13]. The color palette of BL ranges from 400 to 560 nm [14]. Recently, Tamaki et al. have developed new CTZ analogues to cover blue to near-infrared (NIR) in the palette [15].

Some of the CTZ analogues have been developed not only for luminescing with natural marine luciferases but also with Artificial luciferases (ALucs), which were fabricated by extracting frequently occurring amino acids from the alignment of copepod luciferases in public databases [16,17]. This CTZ–ALuc imaging system has been utilized to develop a series of single-chain BL imaging probes for visualizing the activities of steroid hormones and small bioactive molecules [16,18].

When it comes to bioassays and molecular imaging, the multiplex imaging property should be a key breakthrough considering the complexity of molecular events in living subjects. Nevertheless, most imaging systems have depended on 1-dimensional optical intensity alone. Moreover, BL spectra of conventional reporter luciferases have broad bandwidths and are almost superimposed on each other. Any multiplex imaging systems with luciferases are significantly hampered by optical contamination, which cannot be fundamentally overcome by conventional methods such as spectral unmixing algorisms [19], optical filters [20], and quenching reagents [21].

This issue was partly addressed by a series of unique CTZ analogues which exclusively react with a specific reporter luciferase among multiple reporters [22,23]. Specific reporter tagging systems such as Halo-tag have the potential to address such optical contaminations among multi-reporters [24]. Alternatively, a multiple reporter-gene assay system may be a breakthrough to tackle such optical contamination between reporters [25]. However, this method also has common demerits, such as the non-specific background drifts by basial organism activities and a relatively long stimulation time by ligands until the reporters are accumulated enough to be determined by spectrophotometers.

In this study, we introduce a unique combinatorial bioluminescence imaging system, which simultaneously reports multiple BL indexes, i.e., BL intensity patterns and colors as the signatures with novel CTZ analogues named “S-series”. For this study, we synthesized ten kinds of S-series CTZ analogues and characterized the BL intensities, spectra, BL intensity patterns, and chemical stability.

In this study, we represent that the S-series CTZ analogues, **S1**, **S5**, **S6**, and **S7** commonly generate unique, red-shifted BL spectra in green to orange with major marine luciferases, RLuc, NLuc, and ALuc. The colors complete the overall visible region in the BL color palette when combined with the violet and blue of our previous C-series CTZ analogues [26]. The selected CTZ analogues, **S1**, **S5**, **S6**, and **C6**, luminesce distinctive luciferase specificity with each other, and thus collectively create unique BL intensity patterns, such as a signature according to the applied luciferases, R86SG, NLuc, ALuc16, and ALuc47. The dual color BL spectra of RLuc8.6-535SG (R86SG), ALuc16, and NLuc in multi-reporter systems are efficiently unmixed with the virtue of the specificities of the substrates. The utility of this BL imaging system is further demonstrated with various single-chain molecular tension probe systems embedding NLuc and ALuc16, which show that the imaging system simultaneously reported three different optical indexes; unique BL intensity and color patterns identify the reporters, besides the ligand-specific fold intensities in mammalian cells. This study directs a new combinatorial BL imaging system to specifically image molecular events with multiple optical indexes in mammalian cells.

## 2. Results and Discussion

### 2.1. Molecular Designs of Novel S-Series CTZ Analogues (***S1***–***S10***)

**Figure 1:**
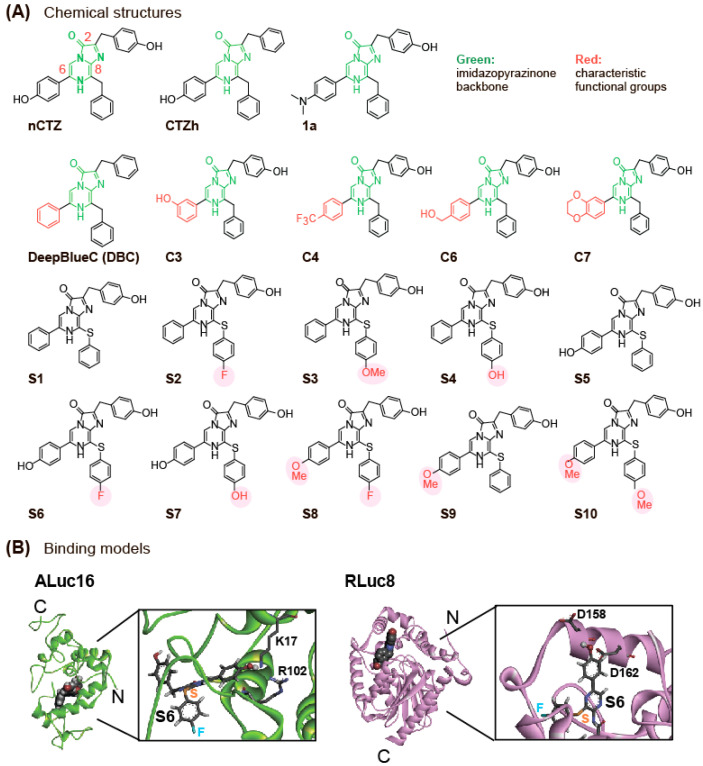
(**A**) Chemical structures of S-series CTZ analogues, together with conventional ones, were used in this study. The imidazopyrazinone backbone is highlighted in green. The characteristic functional groups are shadowed in red. Abbreviations are as follows: nCTZ, native coelenterazine; CTZh, coelenterazine h; DBC, DeepBlueC. The structures of **1a**, **C3**, **C4**, **C6**, and **C7** were obtained from our precedent studies [15,26]. (**B**) Putative three-dimensional (3-D) structures of ALuc16 and *Renilla* luciferase 8 (RLuc8) when they bind with **S6**. These structures were modeled based on the previous study [26] (with the D162A residue mutated back to wild type), and each substrate was manually modified to be **S6**. Putative interacting residues are represented by sticks. Here, the structural modeling of NLuc was not performed because its catalytic site still has many uncertainties [29].

In this study, new CTZ analogues (named **S1**–**S10**) were synthesized by modifying the functional groups at the C-6 and C-8 positions of the imidazopyrazinone backbone (Figure 1). The substrate designs were inspired by a precedent study on the X-ray crystallographic information of RLuc8 and NLuc (Protein Data Bank ID: 5IBO) [27,28], where the active sites closely interact with the C-6 and C-8 positions of nCTZ. Therefore, the C-6 and C-8 positions of nCTZ were modified under the expectation of the specificity, color variation, and enhanced BL intensity with marine luciferases. In the molecular design, the carbon ankle of the benzyl group at the C-8 position was replaced with sulfur (S) for extending the π-electron conjugation. In addition, the *p*-position of the benzyl structure at the C-8 position was modified with various functional groups such as fluorine (F), methoxy (OMe), and hydroxyl (OH) groups (Figure 1). The synthesized substrates (**S1**–**S10**) were named “S-series” CTZ analogues. These S-series CTZ analogues are structurally distinctive from our precedent C-series CTZ analogues, which were characteristic in the modification at the C-6 positions [26].

### 2.2. Characterization of S-Series CTZ Analogues ***S1***–***S10***

**Figure 2:**
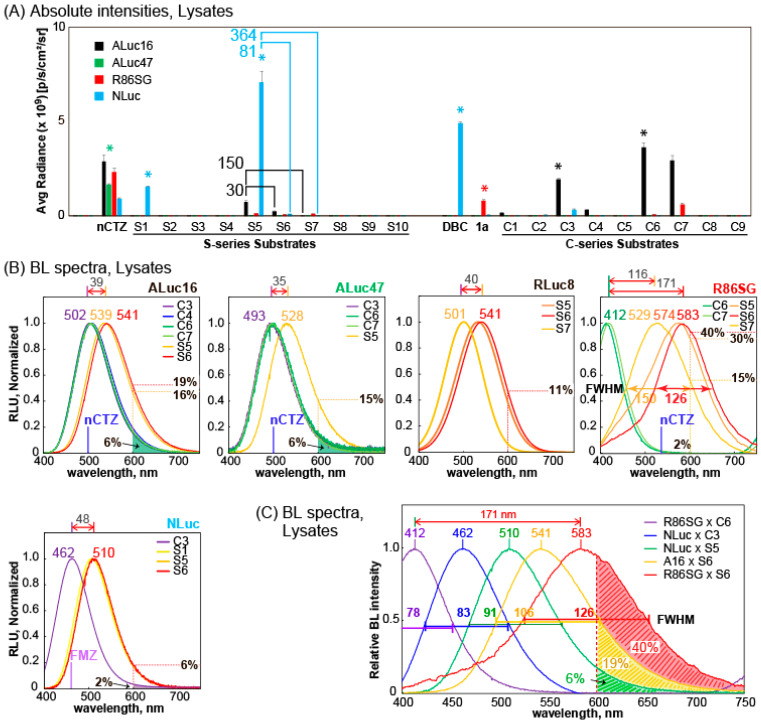
Characterization of S- and C-series CTZ analogues in the presence of marine luciferases. (**A)** Comparison of absolute BL intensities of the S- and C-series CTZ analogues according to marine luciferases, ALuc16, ALuc47, R86SG, and NLuc. The asterisks denote characteristic BL signals. (**B**) The representative BL spectra of selected CTZ analogues in the presence of ALuc16, ALuc47, R86SG, and NLuc. (**C**) Normalized BL spectra of selected S- and C-series showing various colors covering all the visible regions. The numbers at the peaks denote the wavelengths at the maximal intensities (λ_max_). The full width at half maximum (FWHM) values were specified in the middle of the spectra.

The BL properties of the S-series CTZ analogues **S1**–**S10** together with conventional nCTZ, DBC, **1a**, and C-series **C1**–**C9** were characterized with mammalian COS-7 cells containing ALuc16, ALuc47, R86SG, or NLuc (Figure 2). In this characterization, the CTZ analogue **1a** was selected because it was exclusively bright with RLuc variants in our precedent studies [15,26].

The BL intensity profile showed that the CTZ analogues **S1** and DBC are exclusively bright with NLuc in the lysates. **S5** was the brightest with NLuc and followed by ALuc16 and R86SG. **S6** and **S7** are also luminesced with ALuc16 and R86SG. The previously reported **1a**, **C3**, **C6**, and **C7** also showed their unique specificities to R86SG or ALuc16 [23,26]. In contrast that the CTZ analogues generally exerted biased BL intensities with marine luciferases, nCTZ showed universal reactivities with all the tested marine luciferases without bias.

It is interesting to compare the BL intensities of **S5**, **S6**, and **S7** according to marine luciferases because the only differences between the CTZ analogues are the functional groups at the *p*-position of the benzyl ring at the C-8 position (Figure 1A). In the presence of ALuc47, **S5** showed 30-fold and 150-fold higher intensity than **S6** and **S7**, respectively. A similar tendency was also found with NLuc (Figure 2A). In the presence of NLuc, **S5** was 81-fold and 364-fold brighter than **S6** and **S7**, respectively. However, this tendency was not observed with R86SG. When R86SG exists, the BL intensities of **S5**, **S6**, and **S7** were almost the same. **S1** and **S5** are commonly specific to NLuc, the feature of which is not observed with the other S-series CTZ analogues. The only difference between **S1** and **S5** is the presence or absence of the OH group at the C-6 position: **S1** is solely reactive with NLuc. However, **S5** bearing the OH group recovers its reactivity with other luciferases, although it still exerts the highest BL intensities with NLuc.

The above results may be summarized as follows: (i) the fluorine (F) and hydroxyl (OH) groups at the *p*-position of the benzyl ring at the C-8 position significantly reduce the reactivities with ALuc47 and NLuc but are tolerated by the active site of R86SG; (ii) elimination of the OH group (phenyl alone) at the C-6 position greatly elevates the NLuc specificity; (iii) the methoxy group at the C-6 position worsens the overall reactivity with marine luciferases; and (iv) the phenyl group at the C-8 position is essential for the specificity to NLuc and a modification to the phenyl group decreases the reactivities with NLuc and ALuc47.

### 2.3. BL Spectra of Selected S- and C-Series CTZ Analogues

The BL spectral properties of selected S- and C-series CTZ analogues were determined in the presence of various marine luciferases (Figure 2B,C and Table 1).

ALuc16 generated the BL spectra peaked at ca. 502 nm with **C3**, **C4**, **C6**, and **C7**. The spectral peaks are red-shifted to 539 and 541 nm in the presence of **S5** and **S6** (ca. 39 nm gap), respectively. A similar red-shifted feature was observed with ALuc47 by replacing C-series analogues (**C3**, **C6**, and **C7**) with **S5** (ca. 35-nm gap). NLuc also showed red-shifted BL spectra by simply replacing the substrate **C3** with the S-series ones; the spectral peak of NLuc was found at 462 nm with **C3** and shifted with **S1**, **S5**, or **S6** to ca. 510 nm (ca. 48-nm gap).

The most spectral diversity was found with R86SG. The BL spectrum of R86SG was known to peak at ca. 535 nm with nCTZ [30,31]. In this study, R86SG showed blue-shifted BL spectra peaked at ca. 412 nm with **C6** and **C7**. Conversely, R86SG exerted BL spectra peaked at 574 nm with **S5**, and the BL spectra were red-shifted peaked at 529 and 583 nm with **S7** and **S6**, respectively. The full widths at half maximum (FWHM) values of R86SG–**S5** and R86SG–**S6** combinations were ca. 147 and 126 nm, respectively. There was a noticeable gap (ca. 171 nm) between the peaks of **C6** and **S6**.

It is interesting to discuss the differences in the functional groups of CTZ analogues at C-6 and C-8 positions with respect to the peak wavelength in R86SG. The only difference in **C6** from that in nCTZ is that the hydroxymethyl (-CH_2_OH) group is at the C-6 position. nCTZ is known to show a broad spectrum of emission peaks from blue to green (400–535 nm) because of the four different energy levels of intermediates (i.e., neutral species, amide anion, phenolate anion, and pyrazine anion) [32]. The unique peak at 412 nm with **C6** is attributed to the emission from the neutral intermediate because **C6** does not have the *p*-hydroxyl phenyl group at the C-6 position. Therefore, the phenolate anion form could not be produced; instead, a neutral intermediate may be generated to show a blue-shifted (–123 nm) emission spectrum. On the other hand, the differences in **S6** from nCTZ are the sulfur (S) ankle and the fluoro (F) group at the C-8 position (Figure 1), where F in **S6** has an electron-withdrawing property, whereas S contributes to extending the π-conjugation at the C-8 position. Thus, it is considered that the unique peak value of 583 nm (+48 nm, red-shifted) of **S6** is attributed to the emission from pyrazine anion-like intermediates, although the peak is longer than that of a normal pyrazine anion (530–565 nm).

The overall color variation of the S-series CTZ analogues was demonstrated together with those of C-series ones in Figure 2C. The most blue-shifted and red-shifted BL spectra were observed with R86SG–**C6** and R86SG–**S6** combinations, respectively. The spectra represent violet and yellow-orange colors, and the peak gap is ca. 171 nm (as above). The other colors in the visible region were filled with other combinations, such as NLuc–**C3**, NLuc–**S5**, or ALuc16–**S6** pairs to complete the color palette. In the cases of R86SG–**S6** and ALuc16–**S6** combinations, the spectral portions that are longer than 600 nm were ca. 40% and 19% of the total intensity, respectively. This red-shifted BL is especially advantageous in various imaging cases in biological samples and animal models because it minimizes the absorption and attenuation of physiological ingredients, such as hemoglobin [31]. The overall BL spectral peaks (λ_max_) of selected S- and C-series CTZ analogues were summarized in Table 1.

Collectively, the results may be concluded as follows: (i) S-series CTZ analogues emit commonly more red-shifted BL spectra with marine luciferases than C-series CTZ analogues, (ii) the red-shifted feature of S-series CTZ analogues is contributed by the extended π-conjugation of sulfur at the C-8 position, and (iii) the most red-shifted spectrum of **S6**–R86SG, which peaked at 583 nm is attributed to the emission from a pyrazine anion intermediate.

### 2.4. BL Intensity Profiles of S-Series CTZ Analogues as Signatures to Distinguish Each Marine Luciferase

**Figure 3:**
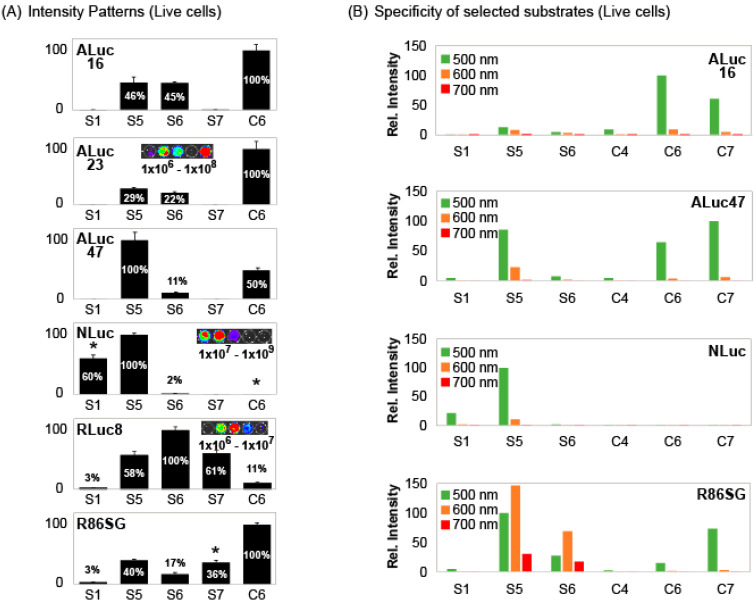
(**A**) BL intensity patterns of selected marine luciferases according to S-series CTZ analogues in living COS-7 cells. The BL intensities of various marine luciferases were normalized to show specific patterns. The asterisks highlight the characteristic features in the BL intensity patterns, different from the others. (**B**) Specification of the colors in the BL intensity patterns according to marine luciferases in living cells. The color intensities specified in the bar graphs were obtained with 500-, 600-, and 700-nm band-pass filters.

The results in Figure 2 inspired us that the BL intensity profiles can be collectively used as unique signatures to identify each luciferase from the others. Therefore, the specific intensity patterns were determined in living cells and summarized in Figure 3A.

The results show that marine luciferases collectively make unique intensity patterns according to the substrates, **S1**, **S5**, **S6**, **S7**, and **C6**, as shown in Figure 3A. For example, **S1** and **S5** were highly bright with NLuc, whereas **S6**, **S7**, and **C6** were very weak in the intensities with it. **S1** is exclusively bright only with NLuc among luciferases.

**S5** appears commonly reactive with all the tested marine luciferases in Figure 3A. However, these appearances are not necessarily exact because Figure 3A is represented in the relative intensities. The absolute intensity profile in Figure 2A shows that **S5** has the best reactivity with NLuc and is followed by ALuc16. This shows that **S5** has its own unique luciferase specificities.

**S7** showed significant BL intensities with RLuc variants (RLuc8 and R86SG), but completely dark with ALuc variants (ALuc16, ALuc23, and ALuc47). Both **S5** and **S6** have almost equal reactivity with ALuc16 and ALuc23, but not with ALuc47 (i.e., **S6** has significantly less reactivity with ALuc47, compared with those with ALuc16 and ALuc23). Thus, **S6** acts as an indicator to distinguish ALuc47 from ALuc16 and ALuc23. It is also interesting that **S6** works as an indicator to discriminate RLuc8 from R86SG because **S6** shows the strongest BL intensity with RLuc8 but one of the weakest with R86SG.

The unique intensity patterns of each luciferase convince us to collectively distinguish each luciferase from the others. These unique intensity patterns were named “signatures” as explained in our very recent study [26].

We examined if the BL intensity patterns are indeed useful for identifying each marine luciferase in unknown samples (Figure S3). The results are as follows: (i) the BL intensity patterns of the well numbers, A1, A4, B2, and C4, are identical to that of NLuc in Figure 3A; (ii) those of the well numbers, A2, B1, B4, and C2, are equal to that of RLuc8 in Figure 3A; and (iii) those of the well numbers, A3, B3, C1, and C3, are the same as that of ALuc23 in Figure 3A. The blind memorandum of Staff 1 exactly matches the above results (predictions) of Staff 2.

The BL intensities in the patterns were further specified with respect to the colors as shown in Figure 3B. When the 500-nm, 600-nm, and 700-nm bandpass filters were applied, the BL intensities showed further biased intensity profiles. Furthermore, when the green filter (the 500-nm BP filter) was applied, **C6** and **C7** showed strong BL intensities with ALuc16. However, **S5** and **S6** lost their BL intensities. It looks very natural because the spectral peak of the **C6**– and **C7**–ALuc16 combinations are at ca. 502 nm (fit to the green filter), but those of **S5**– and **S6**–ALuc16 combinations are at ca. 540 nm. When the green filter was applied, **S5**, **C6** and **C7** retained their BL intensities with ALuc47.

The most dynamic intensity variance was found with R86SG. When the 500-nm and 600-nm filters were applied, **S5** and **S6** retained their significant BL intensities with R86SG because their peaks were at 529 (green) and 583 nm (yellowish orange), respectively, with a broad FWHM as specified above. Conversely, **C6** significantly lost its BL intensity with R86SG because the peak intensity was at 412 nm (violet).

The overall results confirm as follows: (i) optical filters exert biased BL intensity patterns and reflect the colors (peaks) of substrate–luciferase combinations, and (ii) the intensity patterns are also a useful index as optical signatures for identifying each marine luciferase after filtration.

### 2.5. Unmixing of BL Signals and Their Quantitative Relationship in Multi-Reporter Systems

**Figure 4:**
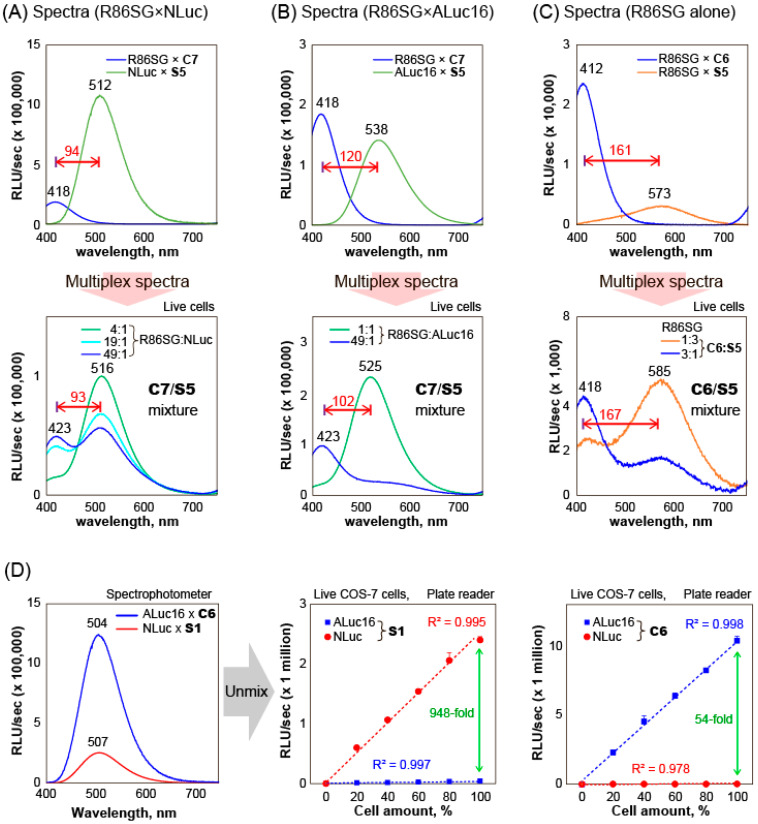
Separation of the BL signals in multiplex reporter systems (**A**) The BL spectra of multiple reporter systems consisting of R86SG and NLuc. The BL spectra were developed by **C7** and **S5**. The upper panel shows respective BL spectra of R86SG and NLuc, and the bottom panel demonstrates the BL spectra of the mixtures of R86SG- and NLuc-containing cells in different ratios. (**B**) The BL spectra of multiple reporter systems consisting of R86SG and ALuc16. The BL spectra were developed by **C7** and **S5**. The upper panel shows respective BL spectra of R86SG and ALuc16, and the bottom panel demonstrates the BL spectra of the mixtures of R86SG- and ALuc16-containing cells in different ratios. (**C**) The BL spectral variation of R86SG according to the substrates, **C6** and **S5**. The upper panel shows respective BL spectra of R86SG with **C6** and **S5**, and the bottom panel demonstrates the BL spectra of R86SG-containing cells that were developed by the mixture of **C6** and **S5**. (**D**) Separation of the BL signals of ALuc16 and NLuc based on the specificity of **C6** and **S1**. The left panel is the BL spectra of ALuc16 and NLuc in the presence of **C6** and **S1**. The left panel reveals that the spectra have completely superimposed each other. The middle and right panels indicate the quantitative feature of the BL signals of COS-7 cells NLuc or ALuc16 in the presence of an access level of **C6** or **S1**. The CTZ analogues **C6** and **S1** selectively luminesce with NLuc and ALuc16, respectively.

The substrate unmixing of BL signals and their quantitative relationship among reporter luciferases was determined by COS-7 cells containing various marine luciferases (Figure 4).

First, the BL spectral separation in multi-reporter systems was examined, as shown in Figure 4A–C. **C7** and **C6** showed largely blue-shifted BL spectra peaked at 418 and 412 nm with R86SG, respectively. On the other hand, in the mixtures, **S5** generated red-shifted BL spectra peaked at 516, 525, and 585 nm with NLuc, ALuc16, and R86SG, respectively. The BL spectral separation allowed us to create a series of multi-reporter systems generating violet, green, and orange colors. In Figure 4A, the mixtures revealed unique BL spectra with two peaks at 423 and 516 nm. The peak heights were almost equivalent when the mixing ratio of the live cells containing R86SG and NLuc is ca. 49:1. The gap between the peaks was found to be ca. 93 nm. Similarly, Figure 4B showed double-peaked BL spectra at 423 and 525 nm. The violet and blue peaks represent the contribution of R86SG and ALuc16, respectively.

In addition, the color variation of R86SG according to the substrates was highlighted in Figure 4C. R86SG showed unique BL spectra peaks at 418 and 585 nm. The peak heights were variable according to the mixing ratios of the substrates, **C6** and **S5**. The peak gap was ca. 167 nm, which is the highest peak separation by the substrates.

The BL spectra of ALuc16 and NLuc were almost superimposed and, thus, impossible to unmix using conventional methods, as shown in Figure 4D. In this study, the substrate-driven unmixing of BL signals was demonstrated using the exclusive specificities of ALuc16–**C6** and NLuc–**S1** combinations and specified the quantitative relationship, where the substrates, **C6** and **S1**, were chosen because they are specifically bright with ALuc16 and NLuc, respectively (Figure 2).

In the measurement, **S1** quantitatively enhanced the BL intensities by increasing the amounts of cells containing NLuc but not with cells containing ALuc16. Their correlation coefficients were ca. 0.995 and 0.997, respectively. On the other hand, **C6** exclusively lifted the BL intensities by increasing the number of cells containing ALuc16 but not with the cells containing NLuc. The correlation coefficients were ca. 0.998 and 0.978, respectively. The graph shows that **C6** emits 54-fold stronger BL intensities with ALuc16 than with NLuc. In contrast, **S1** generates 948-fold brighter BL intensities with NLuc than with ALuc16 at the same 100% cell amount point. These results convince us that **C6** can unmix the BL signal only from ALuc16 in the mixture. Likewise, **S1** can unmix the BL signal from NLuc alone in the mixture. This is called substrate unmixing.

Collectively, the above results confirm the following: (i) the multiple BL signals can be separated by the specific substrates, **S5**, **C6**, and **C7**, in multi-reporter systems; (ii) the unique, double-colored (two-peaked) BL spectra can be created by various combinations of multiple reporter luciferases; and (iii) in the case that the BL spectra of two or more luciferases are completely superimposed, each BL signal can be unmixed by the specificity of the substrates, such as **S1** and **C6**, in a highly quantitative manner.

### 2.6. COS-7 Cell-Based Combinatorial BL Imaging System with Single-Chain Molecular Strain Probes

**Figure 5:**
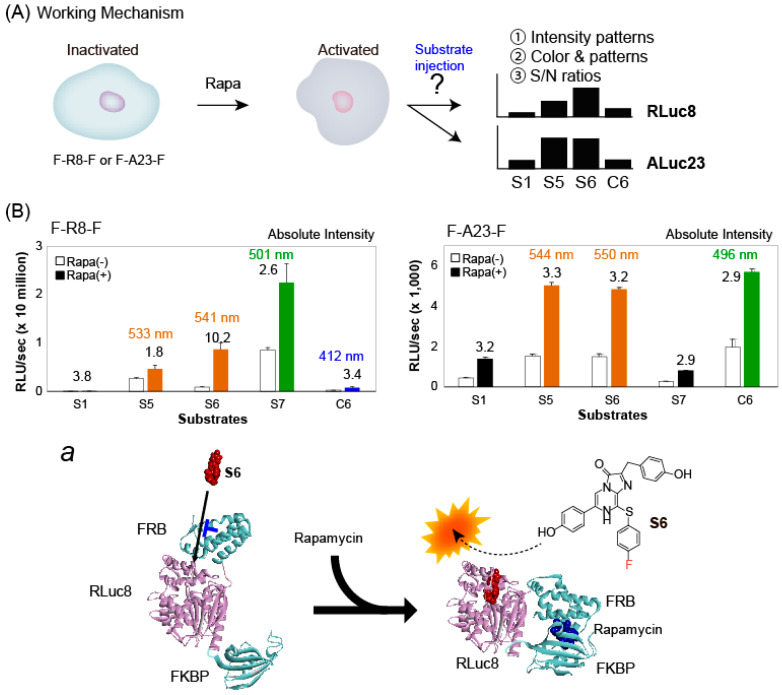
(**A**) Workflow of S-series CTZ-driven assay systems. The cells containing a single-chain molecular probe, F-R8-F or F-A23-F, are stimulated by rapamycin and enhance the BL intensities. The BL intensity patterns (signatures) of F-R8-F, S/N ratios, and the colors according to the substrates are unique. (**B**) The BL intensity patterns (signatures) of F-R8-F (left) and F-A23-F (right) according to the substrates in the presence or absence of rapamycin. The open and closed color bars represent the BL intensities in the absence (-) or presence (+) of rapamycin (rapa), respectively. Inset ***a*** illustrates the model structure of F-R8-F and its working mechanism triggered by rapamycin. The FKBP and FRB structures (with rapamycin) were obtained from the Protein Data Bank (PDB: 1FAP) [33]. The rapamycin-activated FRB–FKBP-binding complex was manually deployed through a consecutive linkage of the components.

The advantages of the S-series CTZ analogues were demonstrated with a multi-index BL assay system for determining rapamycin activities (Figure 5).

As illustrated in Figure 5A, the working mechanism of the assay system is as follows: One or multiple cell lines containing unknown luciferase probes are prepared in blind and stimulated by rapamycin or its vehicle; the cells are then illuminated by multiple S-series CTZ analogues. This assay system provides three different indexes by the addition of specific ligands and substrates. The first index is the BL intensity patterns as the signature, the second is the unique colors in those patterns, and the third is the signal-to-background (S/B) ratios elevated by the ligand.

The results show that the single-chain molecular strain probe, F-R8-F, shows unique BL intensity patterns and dramatic variance in the fold intensities according to the substrates before and after rapamycin stimulation. The overall intensity pattern of F-R8-F roughly reflects that of net RLuc8. The highest intensity of net RLuc8 was obtained with **S6** as shown in Figure 3. In contrast, F-R8-F before rapamycin stimulation emitted one of the lowest intensities with **S6**, as shown in Figure 5B. This may be interpreted as follows: Modifications of the N- and C-terminals of RLuc8, with FRB and FRBP, should modulate both the accessibility of the substrates into the active site and the internal form of the active site; furthermore, every substrate should have distinctive accessibility to the active site and reactivity according to their chemical structures and the functional groups. The result of **S6** suggests that its basal accessibility to the active site may be hampered by FRB and relived after rapamycin stimulation, as illustrated in Figure 5B, inset ***a***. This resulted in the highest fold intensity of 10.2. The second highest fold intensity was obtained by **S1** (i.e., 3.8), which has the simplest substrate structure without any functional groups at the C-6 and C-8 positions among the S-series CTZ analogues. **S7** showed the strongest BL intensities with F-R8-F. However, the fold intensity before and after rapamycin stimulation was merely 2.6. In addition, the BL colors should be another index; that is, **S5** and **S6** generate yellowish green (533 and 541 nm) and **S7** emits green (501 nm), as shown in Table 1.

F-A23-F also showed a unique BL intensity pattern in the presence or absence of rapamycin, and sensitively developed various BL intensities according to the applied substrates. The BL intensity pattern of F-A23-F in the absence of rapamycin was almost equivalent to those of net ALuc23, as shown in Figure 3A. The BL intensities were boosted by rapamycin stimulation, no matter what substrate was applied. The highest fold intensities of F-A23-F were obtained with **S5** and **S6**, which are ca. 3.3 and 3.2, respectively. Upon comparison of absolute intensities, it seems that the net ALuc23 is higher than F-A23-F in the BL intensities. The modification of the N- and C-terminals with other proteins, such as FRB and FKBP, would prevent luminescence. This effect was not observed with F-R8-F. In addition, F-A23-F also generated unique BL color patterns as an index; that is, **S5** and **S6** generate yellowish green (544 and 550 nm) and **C6** generated green (496 nm) (Table 1).

All the results may be summarized as follows: (i) the single-chain molecular strain probes provide three BL indexes for a ligand in assays (i.e., the BL intensity patterns as a signature, the fold intensity, and the colors in pattern with S- and C-series CTZ analogues); (ii) Rapamycin does not induce the color change but varies the intensity patterns of colored bars in the system. The patterns can be used for identifying the presence of rapamycin; and (iii) the present three-index system may be termed a “combinatorial BL imaging system”, where the indexes report three different optical references for a stimulator and minimize potential false positive signals.

### 2.7. Autoluminescence Properties of the S- and C-Series CTZ Analogues

It has been recited that nCTZ and its analogues are decomposed in serum samples and generate autoluminescence. Thus, autoluminescence is a reference to the chemical stability of the substrates. With respect to biocompatibility, it is important to check if the S-series CTZ analogues are decomposed and emit autoluminescence in serum. The autoluminescence of the selected S-series CTZ analogues, **S1**, **S5**, and **S6** together with several C-series CTZ analogues, was determined by using varying concentrations of fetal bovine serum (FBS) (Figure S2).

Among those tested, the highest autoluminescence intensities were determined with **S5** and followed by **S6** and **S1**. The autoluminescent levels are as much as that of CTZh. CTZh, as a conventional substrate, has been broadly applied in various bioassays and animal imaging. Hence, it is likely that the S-series CTZ analogues may be applicable to various physiological samples and animal imaging without severe autoluminescence.

The autoluminescence mechanism of S-series CTZ analogues may be explained with the serum albumin—albumin, taking a major portion of the serum proteins, exposes a lipophobic site to CTZ analogues—which contributes to binding and decomposition of the CTZ analogues [34].

## 3. Materials and Methods

### 3.1. Reagents and Instrumentation for Synthesis of S-Series CTZ Analogues

The starting materials, reagents, and solvents were purchased from Tokyo Chemical Industry Co., Ltd. (Tokyo, Japan), FUJIFILM Wako Pure Chemical Corporation (Osaka, Japan), Kanto Chemical Co., Inc. (Tokyo, Japan) and Sigma-Aldrich (Tokyo, Japan), and used without further purification. Silica gel 70 F254 TLC plates (FUJIFILM Wako, Osaka, Japan) were used for analytical Tin-layer chromatography (TLC), while Silica gel 60 N (spherical, neutral, Kanto Chemical (Tokyo, Japan)) was for column chromatography. For preparative flash chromatography, used was an automated system (Smart Flash EPCLC AI-580S, Yamazen Corp., Osaka, Japan) equipped with universal columns of silica gel. ^1^H and ^13^C NMR spectra were determined with a JEOL ECA-500 instrument (500 MHz for 1 H and 126 MHz for ^13^C) (JEOL, Tokyo, Japan). Mass spectra were obtained with a high-resolution electrospray ionization mass spectrum (JMS-T100LC) (JEOL, Tokyo, Japan) and Matrix-assisted laser desorption/ionization mass spectrum (JMS-S3000 SpiralTOF^TM^-plus 2.0) (JEOL, Tokyo, Japan).

### 3.2. Preparation of Mammalian Cell Expression Vectors Encoding Each Marine Luciferase or Single-Chain Probe

The following plasmids used in this study were obtained from our previous studies: a mammalian expression vector pcDNA3.1(+) (Invitrogen, Waltham, MA, USA) encoding RLuc8, R86SG, NanoLuc (NLuc), ALuc16, ALuc23, or ALuc47 [16,17]. The cDNA templates encoding the FKBP−rapamycin binding domain (FRB) and FK506-binding protein (FKBP) were taken from our precedent study [18]; a pcDNA3.1(+) vector encoded each molecular strain probe, FRB-RLuc8-FKBP (F-R8-F) or FRB-ALuc23-FKBP (F-A23-F) [18]. These single-chain molecular strain probes were made by sandwiching a full-length marine luciferase between FRB and FKBP. After expression, rapamycin triggers an intra-molecular interaction between FRB and FKBP. This interaction appends molecular strain to the sandwiched luciferase and boosts the BL intensity.

### 3.3. Synthesis of S-Series CTZ Analogues

S-series CTZ analogues, **S1**–**S10**, were synthesized according to the following scheme (Figure S1 and Method S1). Compound **2** was synthesized by a Suzuki–Miyaura cross-coupling reaction of commercially available 2-amino-5-bromoaminopyrazine **1** with commercially available Phenylboronic acid and Tetrakis (triphenylphosphine)palladium (0). Compound **3** was synthesized by a similar procedure to compound **2** using Methoxyphenyl boronic acid. We further conducted a bromination using compounds **2** and **3** with commercially available N-Bromosuccinimide to produce compounds **4** and **5**. Compounds **6**–**11** were synthesized from compounds **4** and **5** using Benzenethiol or 4-Methoxybenzenethiol substitution reaction with sodium hydride. Compounds **8**–**11** were then demethylated with boron tribromide to obtain compounds **12**–**15**. Finally, compounds **6**–**15** with ketoacetal derivatives **18** were condensed and cyclized under hydrochloric acid conditions. The synthesized CTZ analogues were named **S1**–**S10**.

The ^1^H-NMR and Mass spectra of S-series CTZ analogues did not show any signals from impurity (Method S1). The spectral information supports that the S-series CTZ analogues are chemically pure enough for the fidelity determination of the BL intensities.

### 3.4. Characterization of Chemical Stability of Selected CTZ Analogues ***S1***, ***S5***, and ***S6***

The autoluminescence of selected CTZ analogues coelenterazine h (CTZh), **S1**, **S5**, **S6**, **C3**, **C4**, **C6**, and **C7** in fetal bovine serum (FBS) samples was determined as a model of the chemical stability in physiological samples (Figure S2).

FBS was diluted with phosphate-buffered saline (PBS) to make 50%, 20%, 10% and 5% dilutions in volume percentage. Forty μL of each dilution were aliquoted into wells of a 96-well black frame microplate. The dilutions in wells were simultaneously injected with 40 μL of the PBS solution containing CTZh, **C3**, **C4**, **C6**, **C7** (final concentration: 50 μM), or vehicle (PBS alone) (named Substrate solution hereafter) using a 12-channel micropipette, where the final concentrations of FBS reached 25% (*v*/*v*), 10% (*v*/*v*), 5% (*v*/*v*) and 2.5% (*v*/*v*). The corresponding BL images of autoluminescence were determined with an IVIS Spectrum imaging system (PerkinElmer) and analyzed with the specific software, Living Image ver. 4.7.

### 3.5. Absolute BL Intensities and Spectra of CTZ Analogues ***S1***–***S10*** According to Marine Luciferases

The absolute BL intensities and spectra of the S-series CTZ analogues, together with our previously reported C-series CTZ analogues, were determined in African green monkey kidney-derived COS-7 cells containing various marine luciferases (Figure 2).

COS-7 cells were grown in 6-well microplates. The cells were transiently transfected with a mammalian expression vector; pcDNA3.1(+) encoded ALuc16, ALuc47, RLuc8, R86SG, or NLuc, and incubated overnight in a humidified 5% (*v*/*v*) CO2 incubator (Sanyo). The cells were harvested by trypsinization and centrifuge and counted using an automatic cell counter (Countess II, Thermo Fisher Scientific, Waltham, MA, USA).

The 104 number of cells were then seeded into the wells of 96-well black-frame microplates for the experiment in Figure 2A. The culture media in the microplates were completely eliminated by suction. The cells in the wells were passively lysed with a lysis buffer (Promega) by immersing the cells with 40 μL of the lysis buffer per well. The wells of cell lysates were simultaneously injected with 40 μL of the Substrate solution dissolving one of the following substrates: nCTZ, CTZh, DeepBlueC (**DBC**), **S1**–**S10**, **C1**–**C9**, and **1a**, using a 12-channel micropipette (final concentration: 50 μM). The microplates were immediately set in the chamber of the IVIS Spectrum imaging system and the consequent BL images were then determined and analyzed with the Living Image ver. 4.7.

Separately, 105 numbers of the cells were seeded into the wells of 12-well microplates for the experiments in Figure 2B,C. The cells were further incubated overnight in the CO_2_ incubator. The culture media in the 12-well microplates were removed by suction. The remained cells on the bottom of each well were then lysed with 200 μL of a lysis buffer (Promega). Forty μL of the cell lysates were then aliquoted into 200-μL PCR tubes. Each tube was injected with 40 μL of the Substrate solution dissolving **S1**, **S5**, **S6**, **S7**, **C3**, **C4**, **C6**, **C7**, or nCTZ (final concentration: 50 μM). The corresponding BL spectra were immediately determined with a high-precision spectrophotometer (AB-1850, ATTO, Tokyo, Japan). This spectrophotometer was used because it can simultaneously acquire all the wavelength lights with high precision and sensitivity. The representative BL spectra were demonstrated according to marine luciferases in Figure 2B,C.

### 3.6. Determination of BL Intensity Patterns of Marine Luciferases According to Selected S- and C-Series CTZ Analogues

The BL intensity patterns were determined in live cells (Figure 3). For the measurements, COS-7 cells were first grown in 6-well microplates. The cells were transiently transfected with a mammalian expression vector; pcDNA3.1(+) encoded ALuc16, ALuc23, ALuc47, NLuc, RLuc8, or R86SG, and incubated overnight in a humidified 5% (*v*/*v*) CO_2_ incubator. The cells were harvested by trypsinization and centrifuge and finally counted using Countess II.

The 5 × 104 number of live cells were then seeded into the wells of 12-well microplates and further incubated overnight in the 5% (*v*/*v*) CO_2_ incubator. After the elimination of the culture media, the cells were harvested by trypsinization and centrifuge and then resuspended with PBS. Twenty mL of the resuspensions were aliquoted into microtubes (ca. 5 × 104 cells per microtube). After injection of 40 μL of the substrate solutions containing **S1**, **S5**, **S6**, **S7**, or **C6** into each microtube (final concentration: 50 μM), the BL intensities were immediately determined with a luminometer (GloMax 20/20, Promega, Madison, WI, USA). The consequent BL intensities in series were analyzed with Excel in Microsoft 365 (Figure 3A). The total BL intensities according to the substrates were collectively compared in patterns as the “BL signatures”.

We further investigated if the BL intensity patterns are indeed useful for identifying each marine luciferase in unknown samples as a blind test (Figure S3). First, Staff 1 grew COS-7 cells in a 12-well microplate to reach 80% confluency. Staff 1 separately prepared three lipofection cocktails using a lipofection reagent (TransIT-LT1, Mirus Bio, Madison, WI, USA), where the cocktails were made by mixing aliquots of the lipofection reagent, serum-free medium, and pcDNA3.1(+) encoding NLuc, RLuc8, or ALuc23 according to the manufacturer’s instruction. 200 μL of each cocktail was randomly injected into each well of the 12-well microplate and incubated overnight. Staff 1 confidentially memorized the injected luciferase names per well and hid them.

Second, Staff 2 took over the 12-well microplate without any information and harvested the cells of each well into separate 1.5-mL microtubes by trypsinization, centrifuge, and resuspension with PBS. Forty μL of the resuspensions were aliquoted into fresh 1.5-mL microtubes and injected with 40 μL of the substrate solutions dissolving **S1**, **S5**, **S6**, **S7**, and **C6** (final concentration: 50 μM). The corresponding BL intensities in serial were immediately determined by the luminometer GloMax 20/20 to determine which luciferases correspond to the unknown samples.

The harvested cells in the above BL intensity pattern study in Figure 3A were further verified with respect to the BL intensities at specific colors in the presence of the CTZ analogues (Figure 3B). The harvested cells were aliquoted into the wells of a flash 96-well black-frame microplate. The wells were then simultaneously injected with various substrate solutions dissolving **S1**, **S5**, **S6**, **C4**, **C6**, or **C7**, using a 12-channel micropipette (final concentration: 50 μM). The BL intensities of the microplate were determined in serial with the IVIS Spectrum imaging system with the 500-nm, 600-nm, and 700-nm bandpass filters. The consequent BL images were then recorded with the Living Image ver. 4.7.

### 3.7. Substrate-Driven Unmixing and Characterization of Multiplex Reporter Systems Based on R86SG, NLuc, and ALuc16

Multiplex reporter signals were unmixed and characterized by diverse combinations of CTZ analogues (Figure 4A–C).

COS-7 cells were grown in 12-well microplates. When the cells reached 70% confluency, they were transiently transfected with a pcDNA3.1(+) vector encoding NLuc, ALuc16, or R86SG and incubated overnight. The cells were then harvested by trypsinization and centrifuge and resuspended in PBS. In the first-round measurement, 50 μL of the resuspensions (5 × 104 cells) were aliquoted into 200-μL PCR tubes. The BL spectra were determined immediately after injection of 50 μL of the substrate solutions dissolving **S1**, **S5**, **C6**, or **C7** using the spectrophotometer AB-1850. In the second-round measurement, the cell resuspension solutions of NLuc, ALuc16, or R86SG were mixed in the indicated ratios: 4:1, 19:1, 49:1, 1:3, or 3:1. Fifty μL of the mixtures were aliquoted into PCR tubes in series and injected with 50 μL of the substrate solutions dissolving **S1**, **S5**, **C6**, or **C7** (final concentration: 50 μM) or with their 25 μL and 25 μL mixtures (final concentration: 25 μM), and the corresponding BL spectra were recorded using the spectrophotometer.

### 3.8. Quantitative Relationship between the Reporters in Multiplex Reporter Systems in Live Cells

The quantitative relationship of the reporters in multiplex reporter systems was characterized by COS-7 cells containing ALuc16 and NLuc (Figure 4D). The BL spectra of COS-7 cells containing ALuc16 or NLuc were determined beforehand according to the same protocol as in Figure 4A–C.

Secondly, a quantitative relationship study was conducted with the harvested COS-7 cells containing ALuc16 or NLuc, which were adjusted to 1 × 10^4^ cell/mL by dilution with PBS (Figure 4D). The cells were further diluted with PBS at different ratios totaling 100%. Forty μL of each dilution were then deployed in the wells of a 96-well black frame microplate. The corresponding BL intensities were determined with a microplate reader (Berthold Technologies, Bad Wildbad, Germany) immediately after the programmed injection of 40 μL of the substrate solutions dissolving **S1** or **C6** (final concentration: 50 μM) and analyzed with Excel 365 (Microsoft Co, WA, USA).

### 3.9. Determination of Rapamycin Activities with Combinatorial Imaging Systems of F-R8-F and F-A23-F in Live Cells

Combinatorial imaging systems were studied with the COS-7 cells expressing F-R8-F or F-A23-F (Figure 5).

COS-7 cells were first cultured in a 12-well microplate and transiently transfected with a pcDNA3.1(+) vector encoding F-R8-F or F-A23-F. The cells were then incubated overnight in the CO_2_ incubator. They were stimulated with 10−6 M rapamycin (final concentration) or its vehicle (0.1% ethanol) and further incubated overnight. The cells with and without rapamycin were separately harvested by trypsinization and centrifuge. The rapamycin-stimulated or -free cells containing F-R8-F or F-A23-F were resuspended with PBS to be 1 × 10^5^ cells/mL. Forty μL of the rapamycin-stimulated and -free cells containing F-R8-F or F-A23-F were separately aliquoted in PCR tubes. The tubes were then injected with various substrate solutions containing **S1**, **S5**, **S6**, **S7**, or **C6** (final concentration: 50 μM). The corresponding BL intensities were determined with the luminometer GloMax 20/20.

## 4. Conclusions

This present study demonstrates a unique combinatorial BL imaging system with novel CTZ analogues named “S-series”. The S-series CTZ analogues were characterized with respect to the BL intensity, color, and specificity of marine luciferases. The results show that the substrates, **S1**, **S5**, **S6**, and **S7**, collectively generate unique color and BL intensity patterns to identify the marine luciferases. The optical signals of the multi-reporter system are efficiently unmixed with the specific substrates. When the system was applied to a single-chain molecular strain probe, one can expect three different optical indexes from the imaging system for a ligand (i.e., unique BL intensity and color patterns identifying the reporters, and the ligand-specific fold intensity in mammalian cells). This study directs a unique methodology on how to construct a combinatorial BL imaging system for specifying molecular events in cells, with their unique BL signatures and intensity profiles.

## Figures and Tables

**Table 1:** The maximal BL wavelengths (λ_max_) of various marine luciferases in the presence of the CTZ analogues, **S1**, **S5**, **S6**, **S7**, **C3**, **C4**, **C6** and **C7.** The bold fonts denote the major peaks.

Luciferase			Substrates (nm)						
	nCTZ	S1	S5	S6	S7	C3	C4	C6	C7
**ALuc16**	496	555	539	541	544	502	508	504	505
**ALuc23**	500		544	550	-	494	-	496	499
**F-A23-F**	500		544	550		499	500	498	499
**ALuc47**	490	502	528	546	-	493	458	502	495
**ALuc49**	490	-	531	-	-	490	465	500	501
**RLuc8**	480		533	541	501	407, 567	-	408	412
**F-R8-F**	490	-	533	541	501	407, 564	-	403	412
**RLuc8.6SG**	535	445	574	583	529	408, 559	415	412	418
**NanoLuc (NLuc)**	481	507	512	510	517	462		465	

## Data Availability

The data presented in this study are available on request from the corresponding author.

## Supplementary Materials

The following supporting information can be downloaded at: https://www.mdpi.com/article/10.3390/ijms24021420/s1.
