# Supplementary material for: S-Series Coelenterazine-Driven Combinatorial Bioluminescence Imaging Systems for Mammalian Cells
Source: Int J Mol Sci. 2023 Jan 11;24(2):1420. doi: 10.3390/ijms24021420 (PMC9865520; doi:10.3390/ijms24021420)
Supplement: Supplementary file 1 [file ijms-24-01420-s001.zip › ijms-2137796-supplementary.pdf]

S-Series Coelenterazine-Driven Combinatorial Bioluminescence Imaging Systems for Mammalian Cells

Contents

1. Synthesis of S-series CTZ analogues (**Figure S1**) page 2

2. The specific procedure of the organic synthesis and the corresponding NMR and Mass data of the S-series CTZ analogues (**Method S1**). page 3

3. Autoluminescent intensities of various CTZ analogues (**Figure S2**) page 9

4. Determination of the BL intensity patterns and colors of unknown luciferases in a 12-well microplate (**Figure S3**) page 10

1. Synthesis of S-series CTZ analogues

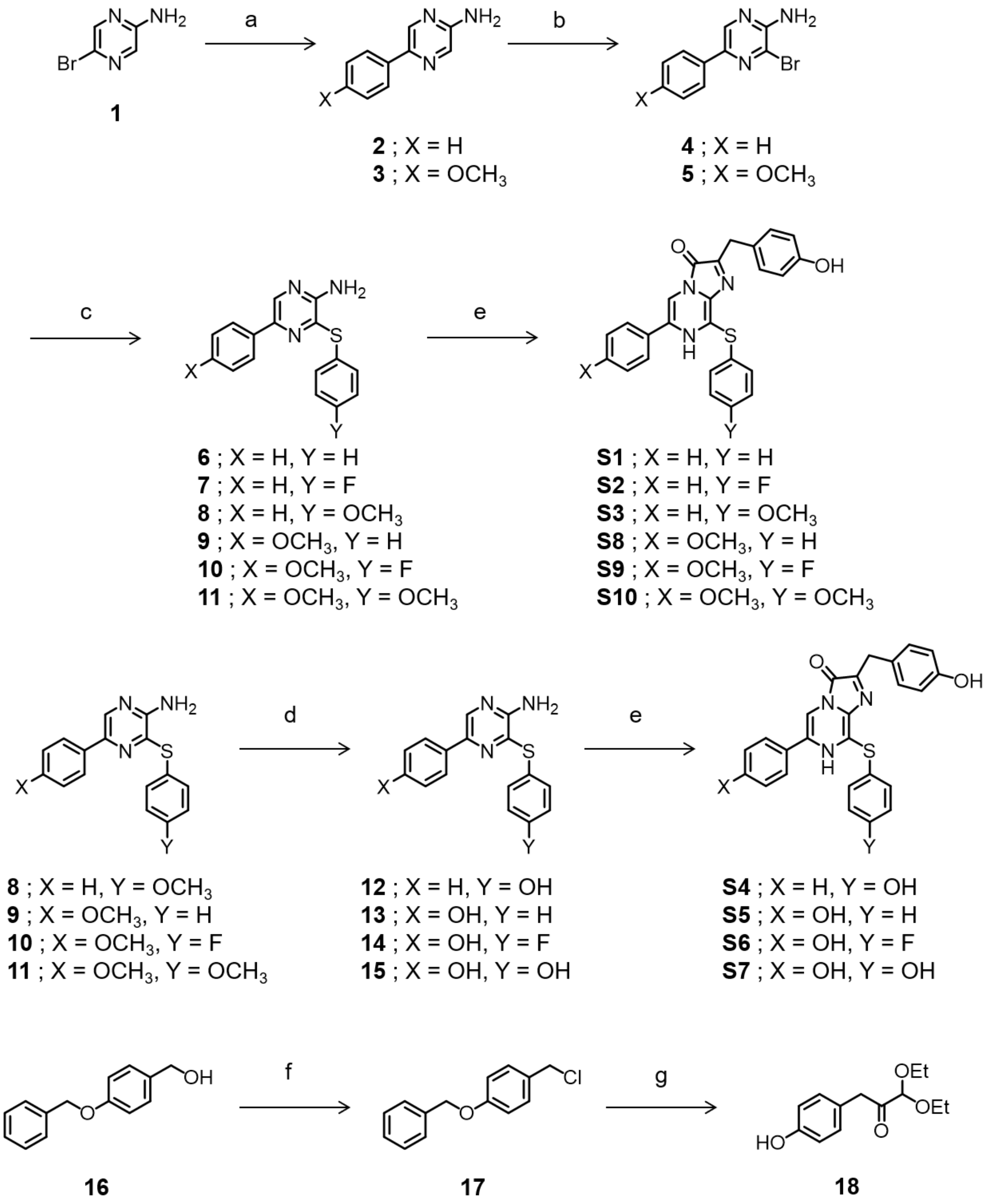

Figure S1. Synthetic scheme for S-series CTZ analogues. Compounds **17** and **18** were synthesized based on our previously reported procedures (Kamiya et al. *Int. J. Mol. Sci.* **2022**, *23*(21), 13047). a: Tetrakis(triphenylphosphine)palladium(0), 1,4-dioxane, 2 M Na_2CO_3 aq, 110 °C. b; *N*-Bromosuccinimide, chloroform, r.t. c; Sodium hydride, *N,N*-dimethylformamide, 0 °C to 110 °C. d; Boron tribromide, chloroform, -80 °C to r.t. e; 12 M HCl aq, eta, 60 °C. f; Thionyl chloride, dichloromethane, 0 °C. g; 1) Mg, 1,2-dibromoethane, ethyl diethoxyacetate, tetrahydrofuran, r.t to reflux to 0 °C to -80 °C. 2) palladium-activated carbon, methanol, hydrogen, r.t.

Method S1. The specific procedure of the organic synthesis and the corresponding NMR and Mass data of the S-series CTZ analogues.

Synthesis of 5-phenylpyrazin-2-amine 2

Compound **1** (1.74 g, 10.0 mmol) and 4-phenylboronic acid (1.34 mg, 11.0 mmol) were dissolved in 1,4-dioxane (40 mL) at room temperature. Tetrakis(triphenylphosphine)palladium(0) (577 mg, 0.055 mmol) and 2 M Na_2CO_3 aq (40 mL) were added to the mixture for 2 hour at 110 °C under Ar. After the reaction was completed, water was added, and the product was extracted with ethyl acetate (2 × 200 mL). The organic phase was dried over anhydrous sodium sulfate and evaporated. The residue was separated by column chromatography (hexane / ethyl acetate = 1/1 to ethyl acetate) to obtain compound **3** (1.57 g, 0.91 mmol, 91%, yellow solid).

^{1}H-NMR (500 MHz, Chloroform-*d*) δ 8.46 (d, J = 1.7 Hz, 1 H), 8.07 (d, J = 1.1 Hz, 1 H), 7.89-7.87 (m, 2 H), 7.47-7.44 (m, 2 H), 7.37 (tt, J = 7.4, 1.3 Hz, 1 H), 4.60 (s, 2 H).
^{13}C-NMR (126 MHz, Acetone-d_6) δ 155.8, 141.6, 139.7, 138.5, 132.4, 129.5, 128.4, 125.9.
HR-MALDI-MS *m*/*z*: $[M+H]^+$ calcd for $C_{10}H_{10}N_3$, 172.08819; found, 172.08692.

Synthesis of 5-(4-methoxyphenyl)pyrazin-2-amine 3

Compound **3** was prepared with a similar procedure to the preparation of **2**.

^{1}H-NMR (500 MHz, Chloroform-*d*) δ 8.40 (d, J = 1.7 Hz, 1 H), 8.04 (d, J = 1.7 Hz, 1 H), 7.81 (td, J = 6.0, 3.4 Hz, 2 H), 6.98 (td, J = 6.0, 3.4 Hz, 2 H), 4.54 (s, 2 H), 3.86 (s, 3 H).
^{13}C-NMR (126 MHz, Chloroform-*d*) δ 159.9, 152.6, 143.1, 138.4, 131.4, 129.7, 126.9, 114.3, 55.4.
HR-ESI-MS: *m*/*z*: $[M+H]^+$ calcd for $C_{16}H_{14}N_3O$, 202.09755; found, 202.09804.

Synthesis of 3-bromo-5-phenylpyrazin-2-amine 4

Compound **2** (1.52 g, 8.87 mmol) was dissolved in chloroform at room temperature. *N*-Bromosuccinimide (2.05 g, 11.5 mmol) was added and the mixture was stirred for 1 h. After the reaction was completed, water was added at 0 °C, and the product was extracted with chloroform (3 × 100 mL). The organic phase was dried over anhydrous sodium sulfate and evaporated. The residue was separated by column chromatography (hexane / ethyl acetate = 3/1 to 2/1) to obtain compound **4** (1.03 g, 4.14 mmol, 46%, reddish brown solid).
^{1}H-NMR (500 MHz, Chloroform-*d*) δ 8.41 (s, 1H), 7.87 (dt, J = 6.9, 1.4 Hz, 2H), 7.47-7.43 (m, 2H), 7.37 (tt, J = 7.4, 1.5 Hz, 1H), 5.06 (s, 2H).
^{13}C-NMR (126 MHz, Chloroform-*d*) δ 151.2, 143.2, 137.8, 135.5, 128.9, 128.6, 125.8, 125.7.

HR-ESI-MS: *m/z*: $[M+H]^+$ calcd for $C_{10}H_9{}^{79}BrN_3$, 249.99900; found, 249.99798 HR-ESI-MS: *m/z*: $[M+H]^+$ calcd for $C_{10}H_9{}^{81}BrN_3$, 251.99601; found, 251.99594.

Synthesis of 3-bromo-5-(4-methoxyphenyl)pyrazin-2-amine 5

Compound **5** was prepared with a similar procedure to the preparation of **4**.

^{1}H-NMR (500 MHz, Chloroform-*d*) δ 8.34 (s, 1H), 7.81 (td, J = 6.0, 3.4 Hz, 2H), 6.97 (td, J = 6.0, 3.4 Hz, 2H), 4.99 (s, 2H), 3.85 (s, 3H).
^{13}C-NMR (126 MHz, Chloroform-*d*) δ 160.2, 150.7, 143.3, 137.1, 128.2, 127.1, 125.7, 114.3, 55.4.
HR-ESI-MS: *m/z*: $[M+H]^+$ calcd for $C_{11}H_{11}{}^{79}BrN_3O$, 280.00907; found, 280.00855 HR-ESI-MS: *m/z*: $[M+H]^+$ calcd for $C_{11}H_{11}{}^{81}BrN_3O$, 282.00530; found, 251.00650.

Synthesis of 5-phenyl-3-(phenylthio)pyrazin-2-amine 6

Benzenethiol (183 μL, 1.79 mmol) was dissolved in dry *N,N*-dimethylformamide (10 mL) and stirred at 0 °C under Ar. Sodium hydride (113 mg, 2.99 mmol) was added to the mixture, and the mixture was stirred for 1 h at 0 °C under Ar. Compound **4** (300 mg, 1.19 mmol) was added to the mixture, and the mixture was stirred for 1.5 h at 110 °C under Ar. After the reaction was completed, water was added at room temperature, and the product was extracted with ethyl acetate (3 × 50 mL). The organic phase was dried over anhydrous sodium sulfate and evaporated. The residue was separated by column chromatography (hexane / ethyl acetate = 2/1 to 1/1) to obtain compound **6** (224 mg, 0.804 mmol, 67%, brown solid).

^{1}H-NMR (500 MHz, Chloroform-*d*) δ 8.36 (s, 1 H), 7.77 (dd, J = 9.5, 2.0 Hz, 2 H), 7.50 (dt, J = 8.0, 1.9 Hz, 2 H), 7.40-7.36 (m, 5 H), 7.33-7.30 (m, 1 H), 4.91 (s, 2 H).
^{13}C-NMR (126 MHz, Chloroform-*d*) δ 151.6, 143.0, 137.6, 137.0, 136.4, 132.6, 130.5, 129.2, 128.7, 128.2, 128.2, 125.4.
HR-ESI-MS: *m/z*: $[M+H]^+$ calcd for $C_{16}H_{14}N_3S$, 280.09156; found, 280.09084.

Compounds **7-11** were prepared with a similar procedure to the preparation of **6**.

3-((4-Fluorophenyl)thio)-5-phenylpyrazin-2-amine 7

^{1}H-NMR (500 MHz, Chloroform-*d*) δ 8.33 (s, 1 H), 7.73-7.71 (m, 2 H), 7.56-7.53 (m, 2 H), 7.39-7.36 (m, 2 H), 7.33-7.30 (m, 1 H), 7.13-7.10 (m, 2 H), 4.87 (s, 2 H).
^{13}C-NMR (126 MHz, Acetone-*d*$_6$) δ 164.0 (d, J = 247.1 Hz), 152.7, 141.9, 138.7, 137.7,137.4 (d, J = 8.4 Hz), 137.2, 129.5, 128.6, 126.4, 125.8, 117.0 (d, J = 22.8 Hz).
HR-ESI-MS: *m/z*: $[M+H]^+$ calcd for $C_{16}H_{13}FN_3S$, 298.07858; found, 298.08142.

3-((4-Methoxyphenyl)thio)-5-phenylpyrazin-2-amine 8

^{1}H-NMR (500 MHz, Acetone-d_6) δ 8.37 (s, 1 H), 7.78 (dt, J = 8.4, 1.6 Hz, 2 H), 7.55 (td, J = 6.0, 3.4 Hz, 2 H), 7.35-7.25 (m, 3 H), 7.05 (td, J = 5.9, 3.6 Hz, 2 H), 5.87 (s, 1 H), 3.86 (s, 3 H).

^{13}C-NMR (126 MHz, Chloroform-*d*) δ 160.3, 150.8, 135.9, 135.8, 128.9, 128.7, 128.1, 126.0, 125.4, 120.0, 115.1, 114.8, 55.4.

HR-ESI-MS: *m*/*z*: $[M+H]^+$ calcd for $C_{17}H_{16}N_3OS$, 310.10031; found, 310.10141.

5-(4-Methoxyphenyl)-3-(phenylthio)pyrazin-2-amine 9

^{1}H-NMR (500 MHz, Chloroform-*d*) δ 8.30 (s, 1 H), 7.71 (dd, J = 6.6, 2.0 Hz, 2 H), 7.49 (dd, J = 8.3, 1.4 Hz, 2 H), 7.40-7.35 (m, 3 H), 6.91 (dd, J = 6.9, 2.3 Hz, 2 H), 4.84 (s, 2 H), 3.83 (s, 3 H).

^{13}C-NMR (126 MHz, Chloroform-*d*) δ 160.0, 151.3, 143.1, 137.4, 136.5, 132.6, 130.8, 129.5, 129.3, 128.2, 126.8, 114.2, 55.4.

HR-ESI-MS: *m*/*z*: $[M+H]^+$ calcd for $C_{17}H_{16}N_3OS$, 310.10158; found, 310.10141.

3-((4-Fluorophenyl)thio)-5-(4-methoxyphenyl)pyrazin-2-amine 10

^{1}H-NMR (500 MHz, Chloroform-*d*) δ 8.26 (s, 1 H), 7.65 (dd, J = 6.9, 2.3 Hz, 2 H), 7.54-7.51 (m, 2 H), 7.12-7.08 (m, 2 H), 6.89 (dd, J = 6.9, 1.7 Hz, 2 H), 4.89 (s, 2 H), 3.82 (s, 3 H).

^{13}C-NMR (126 MHz, Acetone-d_6) δ 163.9 (d, J = 247.1 H), 160.7, 152.2, 142.2, 138.3, 137.3 (d, J = 8.4 Hz,), 136.5, 130.3, 127.1, 126.6, 117.0 (d, J = 22.8 Hz), 114.9, 55.6.

HR-ESI-MS: *m*/*z*: [M+H]+ calcd for $C_{17}H_{15}FN_3OS$, 328.09432; found, 328.09199.

5-(4-Methoxyphenyl)-3-((4-methoxyphenyl)thio)pyrazin-2-amine 11

^{1}H-NMR (500 MHz, Chloroform-*d*) δ 8.23 (s, 1 H), 7.68-7.66 (m, 2 H), 7.51-7.48 (m, 2 H), 6.94 (dd, J = 6.6, 2.0 Hz, 2 H), 6.90-6.88 (m, 2 H), 4.79 (s, 2 H), 3.85 (s, 3 H), 3.82 (s, 3 H).

^{13}C-NMR (126 MHz, Chloroform-*d*) δ 160.2, 159.8, 150.3, 142.7, 139.2, 135.7, 135.3, 129.3, 126.6, 120.2, 114.8, 114.1, 55.4, 55.3.

HR-ESI-MS: *m*/*z*: $[M+H]^+$ calcd for $C_{18}H_{18}N_3O_2S$, 340.11221; found, 340.11197.

Synthesis of 4-((3-amino-6-phenylpyrazin-2-yl)thio)phenol 12

Compound **8** (100 mg, 0.32 mmol) was dissolved in dehydrated dichloromethane (10 mL) and stirred at -80 °C under Ar. Boron tribromide (17% in dichloromethane, 1 moL/L) (2.42 mL, 2.42 mmol) was added to the mixture at -80 °C, and the mixture was stirred for 12 hours at room temperature under Ar. After the reaction was completed, saturated $NaHCO_3$ aq was added at 0 °C, and the product was extracted with chloroform (3 × 50 mL). The organic phase was dried over anhydrous sodium sulfate and evaporated. The residue was separated by

column chromatography (hexane / ethyl acetate = 3/1 to 1/1) to obtain compound **12** (74 mg, 0.25 mmol, 78%, yellow solid).

^{1}H-NMR (500 MHz, Acetone-d_6) δ 8.35 (s, 1 H), 7.80-7.77 (m, 2 H), 7.46 (q, J = 2.9 Hz, 2 H), 7.35-7.32 (m, 2 H), 7.28-7.25 (m, 1 H), 6.95 (q, J = 2.9 Hz, 2 H).
^{13}C-NMR (126 MHz, Acetone-d_6) δ 159.4, 152.3, 141.8, 140.3, 137.9, 137.6, 136.4, 129.4, 128.5, 125.8, 119.2, 117.1.
HR-ESI-MS: *m*/*z*: $[M+H]^+$ calcd for $C_{16}H_{14}N_3O_2S$, 296.08546; found, 296.08576.

Compounds **13-15** were prepared with a similar procedure to the preparation of **12**.

4-(5-Amino-6-(phenylthio)pyrazin-2-yl)phenol 13
^{1}H-NMR (500 MHz, Acetone-d_6) δ 8.31 (s, 1 H), 7.64 (dd, J = 6.9, 2.3 Hz, 2 H), 7.51 (dt, J = 8.2, 1.9 Hz, 2 H), 7.42-7.35 (m, 3 H), 6.80 (dd, J = 6.6, 2.0 Hz, 2 H), 5.75 (s, 2 H).
^{13}C-NMR (126 MHz, Acetone-d_6) δ 158.5, 152.7, 142.7, 137.5, 136.9, 133.9, 132.0, 130.0, 129.3, 129.0, 127.3, 116.3.
HR-ESI-MS: *m*/*z*: $[M+H]^+$ calcd for $C_{16}H_{14}N_3OS$, 296.08613; found, 296.08576.

4-(5-Amino-6-((4-fluorophenyl)thio)pyrazin-2-yl)phenol 14
^{1}H-NMR (500 MHz, Methanol-d_4) δ 8.15 (s, 1 H), 7.57 (td, J = 5.9, 2.5 Hz, 2 H), 7.50 (dd, J = 6.6, 2.0 Hz, 2 H), 7.20-7.16 (m, 2 H), 6.73 (dd, J = 6.6, 2.0 Hz, 2 H).
^{13}C-NMR (126 MHz, Methanol-d_4) δ 163.3 (d, J = 248.3 Hz), 158.9, 152.1, 143.3, 139.9, 137.8 (d, J = 8.4 Hz), 135.6, 129.4, 127.5, 126.8, 116.4, 117.2 (d, J = 22.8 Hz).
HR-ESI-MS: *m*/*z*: $[M+H]^+$ calcd for $C_{16}H_{13}FN_3OS$, 314.07754; found, 314.07634.

4-(5-Amino-6-((4-hydroxyphenyl)thio)pyrazin-2-yl)phenol 15
^{1}H-NMR (500 MHz, Methanol-d_4) δ 8.10 (s, 1 H), 7.52 (dd, J = 6.9, 2.3 Hz, 2 H), 7.40 (dd, J = 6.6, 2.0 Hz, 2 H), 6.87 (dd, J = 6.6, 2.0 Hz, 2 H), 6.72 (dd, J = 6.6, 2.0 Hz, 2 H)
^{13}C-NMR (126 MHz, Acetone-d_6) δ 159.3, 158.4, 151.7, 142.4, 139.8, 137.4, 135.5, 129.5, 127.3, 119.5, 117.1, 116.2.
HR-ESI-MS: *m*/*z*: $[M+H]^+$ calcd for $C_{16}H_{14}N_3O_2S$, 312.08156; found, 312.08067.

Synthesis of 2-(4-hydroxybenzyl)-6-phenyl-8-(phenylthio)imidazo[1,2-a]pyrazin-3(7H)-one S1

Compound **6** (30 mg, 0.11 mmol) and compound **13** (38 mg, 0.16 mmol) were dissolved in ethanol at room temperature. 12 M HCl aq (100 μL) was added to the mixture for 12 h at

60 °C under Ar. The mixture was evaporated. The residue was separated by automated flash chromatography (Smart Flash EPCLC AI-580S, Universal Columns, chloroform /methanol = 99/1 to 15/85) to obtain analogue **S1** (18 mg, 0.044 mmol, 41 %, reddish—brown solid).

^{1}H-NMR (500 MHz, Methanol-*d*$_4$) *δ* 8.45 (s, 1H), 7.72-7.69 (m, 4 H), 7.56-7.53 (m, 3 H), 7.32 (dd, *J* = 3.7, 2.6 Hz, 3 H), 7.12 (dd, *J* = 11.5, 2.9 Hz, 2 H), 6.74 (dd, *J* = 6.6, 2.0 Hz, 2 H), 4.11 (s, 2 H).

HR-ESI-MS: *m/z*: [M+H]$^+$ calcd for $C_{25}H_{20}N_3O_2S$, 426.12772; found, 426.12762.

Compounds **S2**-**S10** were prepared with a similar procedure to the preparation of **S1**.

8-((4-Fluorophenyl)thio)-2-(4-hydroxybenzyl)-6-phenylimidazo[1,2-a]pyrazin-3(7H)-one S2

^{1}H-NMR (500 MHz, Methanol-*d*$_4$) *δ* 8.24 (s, 1 H), 7.69-7.63 (m, 4 H), 7.31-7.22 (m, 5 H), 7.12 (d, J = 8.6 Hz, 2 H), 6.70 (dd, J = 6.6, 2.0 Hz, 2 H), 4.03 (s, 2 H).

HR-ESI-MS: *m/z*: [M+H]$^+$ calcd for $C_{25}H_{19}FN_3O_2S$, 444.11817; found, 444.11817

2-(4-Hydroxybenzyl)-8-((4-methoxyphenyl)thio)-6-phenylimidazo[1,2-a]pyrazin-3(7H)-one S3

1H-NMR (500 MHz, Methanol-*d*$_4$) *δ* 8.29 (s, 1 H), 7.72 (dd, J = 7.7, 1.4 Hz, 2 H), 7.59 (dd, J = 6.6, 2.0 Hz, 2 H), 7.33-7.29 (m, 3 H), 7.12 (d, J = 8.6 Hz, 2 H), 7.07 (dd, J = 6.6, 2.0 Hz, 2 H), 6.70 (dd, J = 6.6, 2.0 Hz, 2 H), 4.04 (s, 2 H), 3.88 (s, 3 H).

HR-ESI-MS: *m/z*: [M+H]$^+$ calcd for $C_{26}H_{22}N_3O_3S$, 456.13864; found, 456.13819.

2-(4-Hydroxybenzyl)-8-((4-hydroxyphenyl)thio)-6-phenylimidazo[1,2-a]pyrazin-3(7H)-one S4

^{1}H-NMR (500 MHz, Methanol-*d*$_4$) *δ* 8.34 (s, 1 H), 7.73 (d, J = 8.0 Hz, 2 H), 7.48 (d, J = 6.9 Hz, 2 H), 7.34-7.28 (m, 4 H), 7.10 (d, J = 8.6 Hz, 2 H), 6.92 (d, J = 8.0 Hz, 2 H), 6.71 (d, J = 6.9 Hz, 2 H), 4.05 (s, 2 H)

HR-ESI-MS: *m/z*: [M+H]$^+$ calcd for $C_{25}H_{20}N_3O_3S$, 442.12175; found, 442.12254.

2-(4-Hydroxybenzyl)-6-(4-hydroxyphenyl)-8-(phenylthio)imidazo[1,2-a]pyrazin-3(7H)-one S5

^{1}H-NMR (500 MHz, Methanol-*d*$_4$) *δ* 8.16 (s, 1 H), 7.66 (dd, J = 7.2, 2.0 Hz, 2 H), 7.53- 7.49 (m, 5 H), 7.11 (d, J = 8.6 Hz, 2 H), 6.72 (d, J = 4.6 Hz, 2 H), 6.70 (d, J = 4.0 Hz, 2 H), 4.03 (s, 2 H).

HR-ESI-MS: *m/z*: [M+H]$^+$ calcd for $C_{25}H_{20}N_3O_3S$, 442.12153; found, 442.12254.

8-((4-fluorophenyl)thio)-2-(4-hydroxybenzyl)-6-(4-hydroxyphenyl)imidazo[1,2-a]pyrazin-3(7H)-one S6

^{1}H-NMR (500 MHz, Methanol-*d*$_4$) δ 8.10 (s, 1 H), 7.65 (qd, J = 5.7, 3.0 Hz, 2 H), 7.46 (dd, J = 6.9, 2.3 Hz, 2 H), 7.24-7.21 (m, 2 H), 7.10 (d, J = 8.0 Hz, 2 H), 6.71 (d, J = 8.6 Hz, 2 H), 6.69 (dd, J = 6.6, 2.0 Hz, 2 H), 4.02 (s, 2 H).

HR-ESI-MS: *m/z*: $[M+H]^+$ calcd for $C_{25}H_{19}FN_3O_3S$, 460.11304; found, 460.11311.

2-(4-Hydroxybenzyl)-6-(4-hydroxyphenyl)-8-((4-hydroxyphenyl)thio)imidazo[1,2-a]pyrazin-3(7H)-one S7

^{1}H-NMR (500 MHz, Methanol-*d*$_4$) δ 8.08-8.14 (s, 1 H), 7.51 (d, J = 8.6 Hz, 2 H), 7.47- 7.44 (m, 2 H), 7.11 (d, J = 8.0 Hz, 2 H), 6.91 (d, J = 8.6 Hz, 2 H), 6.72 (d, J = 9.2 Hz, 2 H), 6.70 (d, J = 8.6 Hz, 2 H), 4.02 (s, 2 H).

HR-ESI-MS: *m/z*: $[M+H]^+$ calcd for $C_{25}H_{20}N_3O_4S$, 458.11649; found, 458.11745.

2-(4-Hydroxybenzyl)-6-(4-methoxyphenyl)-8-(phenylthio)imidazo[1,2-a]pyrazin-3(7H)-one S8

^{1}H-NMR (500 MHz, Methanol-*d*$_4$) δ 8.10 (s, 1 H), 7.61 (d, J = 5.2 Hz, 2 H), 7.51-7.46 (m, 5 H), 7.11 (d, J = 8.0 Hz, 2 H), 6.77 (d, J = 7.4 Hz, 2 H), 6.69 (d, J = 8.0 Hz, 2 H), 4.02 (s, 2 H), 3.74 (s, 3 H).

HR-ESI-MS: *m/z*: $[M+H]^+$ calcd for $C_{26}H_{22}N_3O_3S$, 456.13784; found, 456.13819

8-((4-Fluorophenyl)thio)-2-(4-hydroxybenzyl)-6-(4-methoxyphenyl)imidazo[1,2-a]pyrazin-3(7H)-one S9

^{1}H-NMR (500 MHz, Methanol-*d*$_4$) δ 8.01 (s, 1 H), 7.54 (qd, J = 5.7, 3.0 Hz, 2 H), 7.41 (d, J = 8.6 Hz, 2 H), 7.12 (t, J = 8.9 Hz, 2 H), 7.00 (d, J = 8.6 Hz, 2 H), 6.70 (d, J = 8.6 Hz, 2 H), 6.59 (d, J = 8.6 Hz, 2 H), 3.91 (s, 2 H), 3.65 (s, 3 H).

HR-ESI-MS: *m/z*: $[M+H]^+$ calcd for $C_{26}H_{21}FN_3O_3S$, 474.12802; found, 474.12876

2-(4-Hydroxybenzyl)-6-(4-methoxyphenyl)-8-((4-methoxyphenyl)thio)imidazo[1,2-a]pyrazin-3(7H)-one S10

^{1}H-NMR (500 MHz, Methanol-*d*$_4$) δ 8.17 (s, 1 H), 7.62 (d, J = 9.2 Hz, 2 H), 7.57 (dd, J = 6.9, 2.3 Hz, 2 H), 7.12 (d, J = 8.0 Hz, 2 H), 7.06 (dd, J = 6.6, 2.0 Hz, 2 H), 6.86 (d, J = 9.2 Hz, 2 H), 6.70 (d, J = 8.6 Hz, 2 H), 4.03 (s, 2 H), 3.88 (s, 3 H), 3.79 (s, 3 H).

HR-ESI-MS: *m/z*: $[M+H]^+$ calcd for $C_{27}H_{24}N_3O_4S$, 486.14819; found, 486.14875.

3. Autoluminescent intensities of various CTZ analogues (Figure S2)

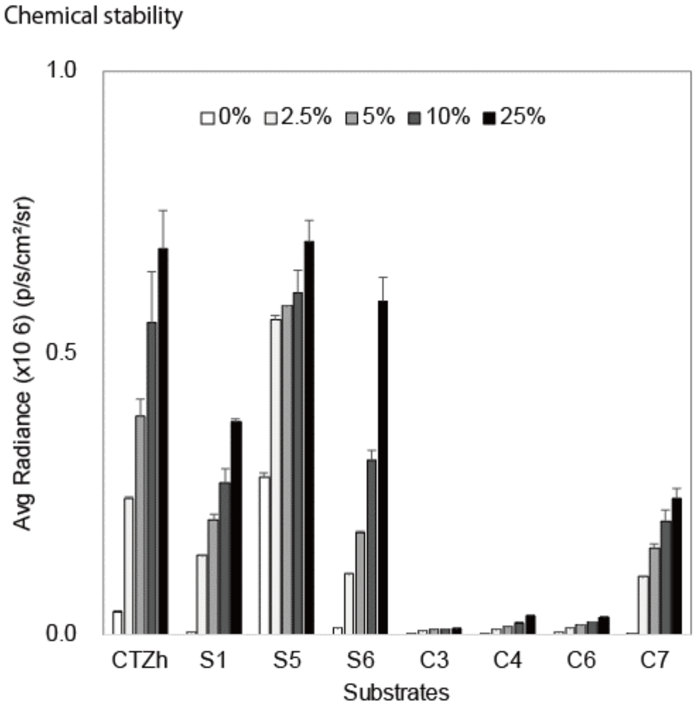

Figure S2. Autoluminescent intensities of various CTZ analogues according to the contents of fetal bovine serum (FBS). The percentage in the annotation denotes the final contents of FBS after the mixture of the substrate solutions.

4. Determination of the BL intensity patterns and colors of unknown luciferases in a 12-well microplate (Figure S3).

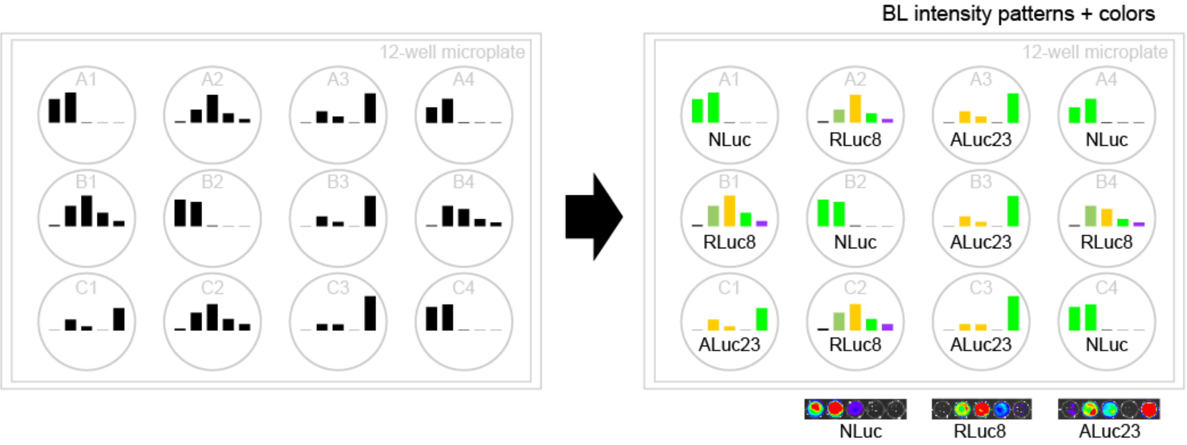

Figure S3. Determination of the BL intensity patterns and colors of unknown luciferases in a 12-well microplate. The BL intensity patterns show unique BL signatures, which implicate what type of luciferase is expressed on each well. The bars inside the patterns have different colors according to which substrate-luciferase combination is applied. The optical image was taken by an IVIS Spectrum imaging system to show the practical BL intensity patterns of each marine luciferase according to a series of CTZ substrates.
